# Supplementary figures and images for: A B-cell actomyosin arc network couples integrin co-stimulation to mechanical force-dependent immune synapse formation
Source: eLife. 2022 Apr 11;11:e72805. doi: 10.7554/eLife.72805 (PMC9142150; doi:10.7554/eLife.72805)

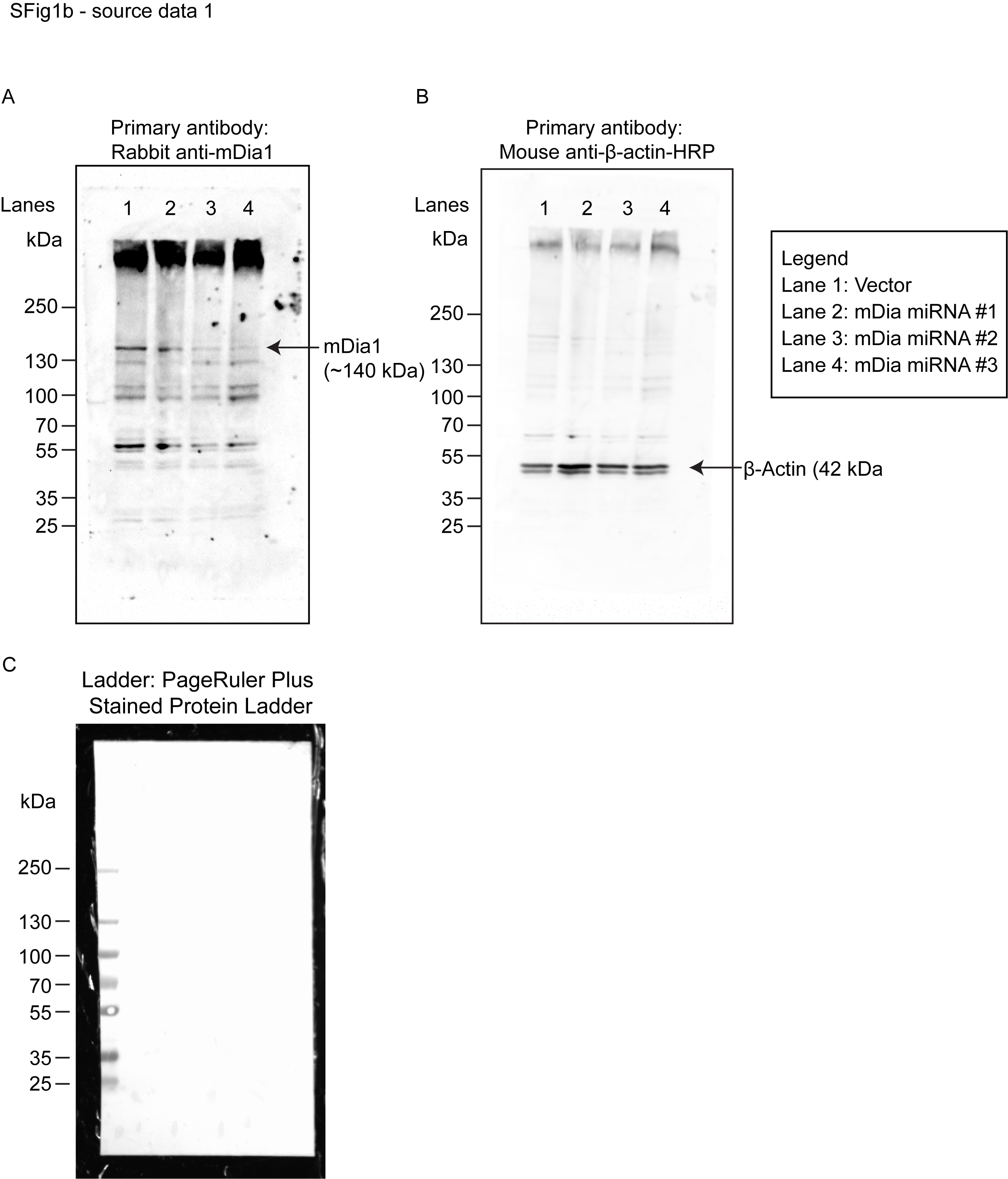

Supplement: Figure 2—figure supplement 1—source data 1. — (A) Entire immunoblot using rabbit anti-mDia1 followed by goat anti-rabbit-HRP secondary. (B) Entire immunoblot using mouse anti-β-actin-HRP. (C) Blot showing the molecular weight ladder Thermo Fisher Page Ruler Plus. Arrows indicate the relevant bands. [file elife-72805-fig2-figsupp1-data1.zip › Raw WB source data for SFig1b-01.jpg]
